# How Negative Social Bias Affects Memory for Faces: An Electrical Neuroimaging Study

**DOI:** 10.1371/journal.pone.0162671

**Published:** 2016-09-21

**Authors:** Alice Mado Proverbio, Francesca La Mastra, Alberto Zani

**Affiliations:** 1 NeuroMi - Milan Center for Neuroscience, Dept. of Psychology, University of Milano-Bicocca, Milan, Italy; 2 Institute of Bioimaging and Molecular Physiology, IBFM-CNR, Milan, Italy; Bournemouth University, UNITED KINGDOM

## Abstract

During social interactions, we make inferences about people’s personal characteristics based on their appearance. These inferences form a potential prejudice that can positively or negatively bias our interaction with them. Not much is known about the effects of negative bias on face perception and the ability to recognize people faces. This ability was investigated by recording event-related potentials (ERPs) from 128 sites in 16 volunteers. In the first session (encoding), they viewed 200 faces associated with a short fictional story that described anecdotal positive or negative characteristics about each person. In the second session (recognition), they underwent an old/new memory test, in which they had to distinguish 100 new faces from the previously shown faces. ERP data relative to the encoding phase showed a larger anterior negativity in response to negatively (vs. positively) biased faces, indicating an additional processing of faces with unpleasant social traits. In the recognition task, ERPs recorded in response to new faces elicited a larger FN400 than to old faces, and to positive than negative faces. Additionally, old faces elicited a larger Old-New parietal response than new faces, in the form of an enlarged late positive (LPC) component. An inverse solution SwLORETA (450–550 ms) indicated that remembering old faces was associated with the activation of right superior frontal gyrus (SFG), left medial temporal gyrus, and right fusiform gyrus. Only negatively connoted faces strongly activated the limbic and parahippocampal areas and the left SFG. A dissociation was found between familiarity (modulated by negative bias) and recollection (distinguishing old from new faces).

## Introduction

Prejudice is a type of "premature judgment" of subjects of which we have no direct, complete and sufficient knowledge. Allport [1] described the prejudice as a feeling, positive or negative, relative to a person or a thing before direct experience or that is not based on the person or thing. Prejudice formation is considered to be an adaptive cognitive process that helps the human mind to process information through the help of categories. After these categories have been created, prejudice inevitably arises. We know that people’s social traits are inferred on the basis of their appearance and that this judgment activates a potential bias able to influence social interactions. At this regard a social context network model (SCNM), a fronto-insular-temporal network has been proposed by Ibáñez and Manes [2], as being responsible for processing social contextual effects, able to update the context and use it to make predictions and consolidate context-target associative learning. Indeed many studies have extensively shown the role of contextual and social factors in in stimulus recognition (e.g., [3]). However, not much is known about the impact of these biases on specific aspects of human cognition, such as memory processes. While walking through the streets of our city, we encounter hundreds of people, and although we will easily forget some of them, others remain etched in our memory. Why are some faces remembered better than others?

A strong indicator of how and when a face is remembered is face typicality [4]. Unusual faces with atypical features are better remembered than more typical faces [5], even if less attractive. Another component that affects facial memory is perceived trustworthiness. Some studies suggest that faces perceived as the most untrustworthy are remembered significantly more than those perceived as reliable [6]. However, in these experiments, characters were perceived as unreliable only on the basis of perceptual appearance. In a study manipulating social traits, Mealey and colleagues [7] associated a face with some fictional personal traits (irrelevant history, history of unreliability, history of cheating) of the person depicted in the photograph. A week later, the experimental subjects were presented the old as well as new faces, and the task was to recognize which one had been previously encountered. The results showed a higher percentage of recognition of persons described as crooks in the learning phase. Generally, negative emotionally-valenced stimuli are remembered better than positive or neutral stimuli. For example, Ochsner [8] reported better performance in an old/new task for faces with a negative facial expression vs. a positive or a neutral face, whereas another study [9], in which both faces and scenes were used as experimental stimuli, showed that greater recognition of stimuli with negative valence occurred compared to those with neutral valence. The reason for this advantage might be due to stronger memory traces for aversive stimuli that might involve associations with amygdala and limbic structures. Indeed, investigations of emotion’s impact on memory have shown that emotion enhances memory [10–12] and that this enhancement is associated with increased engagement of amygdala, hippocampus, parahippocampus, and medial temporal lobe regions [13]. The enhanced activity in these areas has been observed during both encoding [14–17] and retrieval [18–20].

However, a dissociation has been shown between the sensation of knowing something/someone (familiarity) and recall, which is associated with the ability to retrieve the details of the learning situation (where and when that person has been met, for example). This dissociation was first demonstrated by a “remember-know” (R/K) paradigm introduced by Tulving [21]. This paradigm involves a learning phase of a given stimulus material, followed by a memory session in which participants decide whether they "remember" the material and are able to evoke specific qualitative information associated with it or just "know" the stimulus that is familiar to them without the ability to recall specific details. The idea behind this dissociation is that "remembering" reflects the process of recollection, characterized by the conscious recovery of details about the spatiotemporal context associated with stimulus learning. However, "knowing" might be considered an index of stimulus familiarity because it is characterized by the simple sensation of having seen/encountered an item, with no further recovery information related to it [22]. Many ERP studies have provided evidence of a dissociation between *familiarity* and *recollection* [23–28]. Research has shown that recollection is associated with an increased positivity at parietal electrode sites in between 600–900 ms (also known as late positive complex, LPC, or parietal old/new effect). The parietal old-new effect is more substantial for the items recognized as old and correctly associated to their context of study rather than old stimuli that are studied but not properly assigned to their original context of study [29–33]. In contrast, stimulus familiarity is associated with an increased frontal FN400 response (300–600 ms) that would originate in one or more regions of the prefrontal cortex. Its amplitude is reduced or attenuated in response to stimuli already seen or studied; therefore, it is inversely related to stimulus familiarity. The response most likely indicates the coding of the prefrontal cortex for new material.

In an interesting behavioral study, Bell and Buchner [34] showed that 72 faces were accompanied by a description of neutral, negative (disgusting) or positive traits. The study phase was followed by a face recognition task. The results showed that although the old/new discrimination task was not influenced by the type of descriptive information provided with the face (positive, neutral or negative), source memory for faces associated with disgusting behavior (i.e., memory for the disgusting context in which the face was encountered) was consistently better than source memory for other types of faces. The authors concluded that there is a dissociation between recollection and source memory with regard to memory for faces associated with negative or threatening contexts that may be instrumental in avoiding the negative consequences of encounters with persons associated with negative or threatening behaviors. A similar pattern was found a few years earlier in another study by Buchner and coworkers [35].

The aim of the present study was to investigate the effect of a negative social bias on memory for faces. A short fictional story was presented to participants in association with a male or female face in an encoding session; memory recollection of faces was immediately tested in an old/new face recognition paradigm. The effect of memory encoding per se (old vs. new discrimination) was observed by considering ERPs to positively biased faces, whereas the effect of negative prejudice was ascertained by comparing the effects of positive vs. negative bias on memory recognition. A validation pre-test on an independent group of judges showed that both positive and negative biases significantly affected faces trustworthiness perception. Our assumption was that although both positive and negative biases actively modulated face processing, brain response to negative faces would differ from positive faces. We also expected to find a dissociation between the conscious effect of recollection indexed by LP parietal effect (known as old/new parietal effect) and an implicit familiarity effect, dependent on the strength of memory traces and indexed by frontal FN400 modulation.

## Materials and Methods

### Participants

Seventeen right-handed people (6 males and 11 females) aged 22 to 28 years old participated in the study. All participants were right-handed with normal or corrected vision. The handedness was assessed through the administration of the Italian version of the Edinburgh Inventory Questionnaire. We also assessed the lack of any brain damage, previous history of epilepsy or current medications that could affect the electrical activity of the brain. The experiment was conducted with approval from the Ethical Committee of University of Milano-Bicocca and in compliance with APA ethical standards for the treatment of human volunteers (1992, American Psychological Association). Informed written consent was obtained from all subjects. All participants received academic credit for their participation. All experiments were performed in accordance with relevant guidelines and regulations.

### Stimuli

The material consisted of 300 photographs of close-ups of unfamiliar Caucasian-like faces of various ages (150 men and 150 women), collected from various free-photo web sites. Each image had a size of 350 x 438 pixels and was colored and at high resolution. 200 photographs (100 men and 100 women) were used in the encoding task (therefore named “old” faces), whereas the other 100 faces (“new” faces) were used in a subsequent recognition task. In the encoding task, faces were presented in association with a short story (1 sentence) that provided fictional information on the character, such as an anecdote or personal information. As seen from the examples provided in Table 1, the biographic information could be positive, thereby inducing a positive prejudice toward the depicted character or vice versa, a negative prejudice could induce a negative bias. More precisely, 100 photographs (50 men and 50 women) were matched with a positive description relative to personality traits or specific biographical events (POS), and the remaining 100 (50 men and 50 women) were characterized negatively (NEG). The other 100 faces, 50 men and 50 women, were not accompanied by verbal descriptions and were used as novel faces for the memory recognition task (NEW). As displayed in Table 2, faces for the encoding task were carefully balanced by sex, age, facial expression valence across the two categories (negative and positive prejudice). The possible effect of face age, sex or expression on ERPs was not investigated, because it was annulled across classes. Indeed, the aim of the study was to understand the effect of prejudicial bias on face processing, regardless of these variables accurately controlled for.

**Table 1:** Examples of positive and negative descriptions paired to female and male faces of various ages.

CATEGORY	POSITIVE	NEGATIVE
Children	She cries when she sees less fortunate children in TV.	He is just a mischievous child, does not obey anybody.
Adolescent	He defended a classmate being bullied.	He set fire to a girl’s scooter for revenge.
Adult	She gave up chemotherapy to give birth to her son.	Years ago she suffocated her three year old son.
Adult	She is a brilliant American biotechnologist.	She was reported for sexual abuse on her pupils.
Adult	Died during a robbery to protect a child.	Pushes ecstasy tablets in a Milan nightclub.
Adult	He increased the salary of his workers.	He is often absent from work because he gets drunk every night.
Elderly	She prepares delicious cakes for her grandchildren.	She always throws the garbage bags in the river.

**Table 2:** Stimulus randomization and matching of age, sex, facial expression, and valence of prejudice across categories.

Age of faces	Children		Adolescents		YoungAdults		Adults		Elderly		
Facial expression valence	*POS*	*NEG*	*POS*	*NEG*	*POS*	*NEG*	*POS*	*NEG*	*POS*	*NEG*	Total
**Prejudice valence**	**Positive**										
Males	2	2	3	3	8	7	8	7	5	5	50
Females	2	2	3	3	8	7	8	7	5	5	50
**Prejudice valence**	**Negative**										
Males	2	2	3	3	8	7	8	7	5	5	50
Females	2	2	3	3	8	7	8	7	5	5	50
Total stimuli	8	8	12	12	32	28	32	28	20	20	200

The degree of positivity or negativity of verbal description was balanced across the 4 stimulus categories based on the scores obtained in a stimulus validation procedure. Stimuli were validated by administering a social trust test to *20 independent judges (10 women and 10 men ranging in age from 25 to 60 years)*. *Faces were shown to judges for a few seconds*, one by one, using a PowerPoint presentation and accompanied by a short verbal description appearing below. Judges were asked by the experimenter to evaluate the degree of trust inspired by each face by considering the following question: would you trust this person enough to leave your baby nephew or your home keys in their hands? The scale ranged from 1 to 3, from 1 = No, I do not trust this person at all; 2 = I do not know; 3 = Yes, this person inspires confidence.

Using this procedure, 45 faces or descriptions were changed because they were neutral according to at least 75% of judges and were re-tested. The overall scores obtained by each stimulus underwent a repeated measure ANOVA, which demonstrated the efficacy of verbal description in inducing a significantly negative (1.46, SE = 0.04) or positive (2.63, SE = 0.03) bias (F [1,98] = 136, p<0.00001) in both male and female characters (face gender effect was not significant).

Stimulus luminance was individually measured by using a Minolta CS 100 Luminance meter, and a repeated measures ANOVA proved their equiluminance across categories (men POS = 20.56 (SE = 1.19), Men NEG = 21.90 (SE = 1.19), Men NEW = 20.20 (SE = 1.19), Women POS = 21.49 (SE = 1.19), Women NEG = 22.3 (SE = 1.19), Women NEW = 19 (SE = 1.19) Foot-lamberts).

The length (# of words) and number of letters of verbal description of POS and NEG faces was also balanced across classes, as proved by a lack of a statistically significant result (# of words: Men POS = 8.1 (SE = 0.25), Men NEG = 8.08 (SE = 0.25), Women POS = 8.60 (SE = 0.25), Women NEG = 9.14 (SE = 0.25); # of letters: Men POS = 39.56 (SE = 1), Men NEG = 40.44 (SE = 1), Women POS = 43.6 (SE = 1), Women NEG = 44.49 (SE = 1).

### Procedure

**Fig 1:**
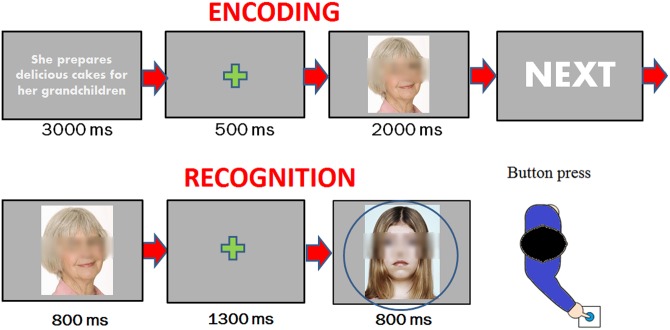
Timing of experimental procedure used in the two conditions: encoding (top) and recognition (bottom) task.

The participants sat in a dark test cubicle that was acoustically and electrically shielded (Faraday cage) in front of a computer screen that was placed at 114 cm from the participants' eyes. The subjects were instructed to gaze at the center of the screen (where a small permanent green cross served as a fixation point) and avoid any eye or body movement during the recording session. The stimuli were presented randomly and mixed (as age, sex, emotional valance, prejudice valance) at the center of the screen in eight different blocks lasting approximately 5 min and preceded by 3 warning signals ‘‘ready”, ‘‘set”, ‘‘go” presented for 500 ms. In the encoding task, sentences were typed in lower case Arial Narrow font and were white on a dark grey background. Each sentence was presented for 3000 ms, and it was followed by a 500 ms interstimulus interval (ISI). Then, the associated face was presented, which lasted for 2000 ms. Each pair of descriptions/face stimuli was followed by the “NEXT” screen that lasted 1 sec (please see Fig 1 for a description of the paradigm) that clearly signaled the presentation of a new pair. In the recognition task, each face was presented for 800 ms with a random ISI varying from 1300–1400 ms. The task consisted of pressing a button with the index finger in response to old faces and with the middle finger in response to new faces as accurately and fast as possible. The left and right hands were used alternately throughout the recording session, and the order of the hand and task conditions were counterbalanced across the subjects. The experiment was preceded by a training phase in which 10 faces and fictional stories that were not included in the experiment were presented. Participants were explicitly told that they had to pay attention to the faces to recall them in the recognition task.

### EEG Recording and Analysis

EEG data were recorded continuously by using the EEProbe system (ANT Software, Enschede, The Netherlands) from 128 scalp sites according to the 10–5 international system [36] at a sampling rate of 512 Hz. Averaged mastoids served as the reference lead. Horizontal and vertical eye movements were also recorded. The EEG and electrooculogram (EOG) were amplified using a half-amplitude band pass of 0.016–100 Hz. Electrode impedance was maintained below 5 kΩ. EEG epochs were synchronized with the onset of the stimulus presentation. Computerized artifact rejection was performed to discard epochs in which eye movements, blinks, excessive muscle potentials, or amplifier blocking occurred. The artifact rejection criterion was a peak-to-peak amplitude that exceeded 50 μV, which resulted in a rejection rate of ~5%. The -100-0 ms time range served as reference for baseline correction. Event-related potentials (ERPs) from 100 ms before (−100 ms) to 1000 ms after face stimuli onset were averaged. ERP epochs associated with behaviorally incorrect responses or with discarded RTs were also rejected. ERP components were identified and measured if they reached their maximum amplitude (and in agreement with previous ERP literature) with respect to the average baseline voltage. The mean area amplitude of ERP components of interest was quantified at the anterior frontal and prefrontal sites (AF7, AF8, FP1, FP2) in the time window 600–800 ms, corresponding to anterior negativity (AN) in the Encoding task. The anterior and inferior frontal sites (AF3, AF4, F5, F6) between 400–600 ms and the parietal sites (P1, P2) between 450–550 ms corresponded to the FN400 and “Old-New” LPC parietal response, respectively, in the recognition task. Multifactorial repeated-measures ANOVAs were applied to the amplitude data. The factors were prejudice valence (POS, NEG) and electrode (anterior frontal, prefrontal) hemisphere (left or right) in the encoding task and face type (POS, NEG, NEW), electrode (varying according to the ERP component, see above) and hemisphere (left, right) in the recognition task. Multiple post-hoc mean comparisons were performed using Tukey's test.

### swLORETA Source Localization

Low-resolution electromagnetic tomography (LORETA) was applied to ERPs recorded during the recognition task for the processing of the 3 types of faces (NEG; POS, NEW) in the 450–550 ms time window. LORETA [37] is a discrete linear solution to the inverse EEG problem and corresponds to the 3D distribution of neuronal electrical activity that has maximally similar (i.e., maximally synchronized) orientation and strength between neighboring neuronal populations (represented by adjacent voxels). In this study, an improved version of the standardized weighted LORETA was used [38]. This version, called swLORETA, incorporates a singular value decomposition-based lead field-weighting method. The source space properties included a grid spacing (the distance between two calculation points) of five points (mm) and an estimated signal-to-noise ratio (which defines the regularization of three; a higher value indicates less regularization and therefore less blurred results) of three. Using a value of 3–4 for the computation of SNR in the Tikhonov's regularization produces superior accuracy of the solutions for any inverse problems. swLORETA was performed on the group data (grand-averaged data) to identify statistically significant electromagnetic dipoles (p<0.05) in which larger magnitudes correlated with more significant activation. The data were automatically re-referenced to the average reference as part of the LORETA analysis. A realistic boundary element model (BEM) was derived from a T1-weighted 3D MRI dataset through segmentation of the brain tissue. This BEM model consisted of one homogeneous compartment comprising 3446 vertices and 6888 triangles. Advanced Source Analysis (ASA) employs a realistic head model of three layers (scalp, skull, and brain) created using the BEM. This realistic head model comprises a set of irregularly shaped boundaries and the conductivity values for the compartments between them. Each boundary is approximated by a number of points, which are interconnected by plane triangles. The triangulation leads to a more or less evenly distributed mesh of triangles as a function of the chosen grid value. A smaller value for the grid spacing results in finer meshes and vice versa. With the aforementioned realistic head model of three layers, the segmentation is assumed to include current generators of brain volume, including both gray and white matter. Scalp, skull, and brain region conductivities were assumed to be 0.33, 0.0042, and 0.33, respectively. The source reconstruction solutions were projected onto the 3D MRI of the Collins brain, which was provided by the Montreal Neurological Institute. At this regard, it should be noted that the realistic head model in LORETA may affect source localization results due to individual differences in head and brain shape. The probabilities of source activation based on Fisher's F-test were provided for each independent EEG source, and their values are indicated by a “unit” scale (the larger, the more significant). Both the segmentation and generation of the head model were performed using the ASA software program Advanced Neuro Technology (ANT, Enschede, Netherlands).

## Results

### Behavioral Results

Reaction times (RTs) that exceeded the mean value ±2 standard deviations were discarded, which resulted in a rejection rate of 2%. Both RTs and hit percentages were subjected to separate multifactorial repeated-measures ANOVAs with one factor of variability: face type (POS, NEG, NEW). The ANOVA performed on RTs yielded the significance of face type (F [2, 22] = 3.949; p<0.03; ε = 0.66; Greenhouse Geyser corrected p = 0.05; η2 = 0.354). Post-hoc comparisons indicated faster responses to OLD than NEW faces (POS: 716 ms, SE = 15; NEG: 723 ms, SE = 17.6; NEW: 750 ms, SE = 17.18), with no effect of bias. The ANOVA performed on hit percentages (old faces correctly recognized as old, or vice versa) also yielded the significance of face type (F [2, 22] = 12.905; p<0.000196; ε = 0.61; Greenhouse Geyser corrected p = 0.00217; η2 = 0.53). Post-hoc comparisons indicated a higher recognition rate (p<0.002) for new than old faces (NEW: 86.55 S.E. = 3.13%; POS = 68.79%, SE = 2.12; NEG: 68.67, SE = 2.15), with no effect of bias. D-prime values (*d’*) were = 1.923 for new faces, 0.911 and 0.908 for positively and negatively biased faces, respectively. Overall prejudice did not significantly affect recollection accuracy.

### Electrophysiological Data

**Fig 2:**
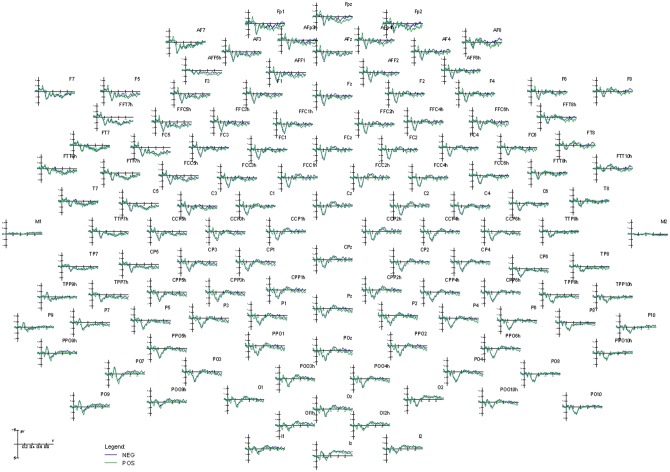
Grand average ERP waveforms recorded from 128 electrode sites during the encoding task. The effect of prejudice affected face processing over prefrontal areas during coding.

The ERPs recorded from high density montage (128 channels) during the encoding task are displayed in Fig 2. It is important to mention that any difference between the two class of ERPs to faces depended on having just read a positive or negative comment on the person depicted.

#### Encoding task

**Fig 3:**
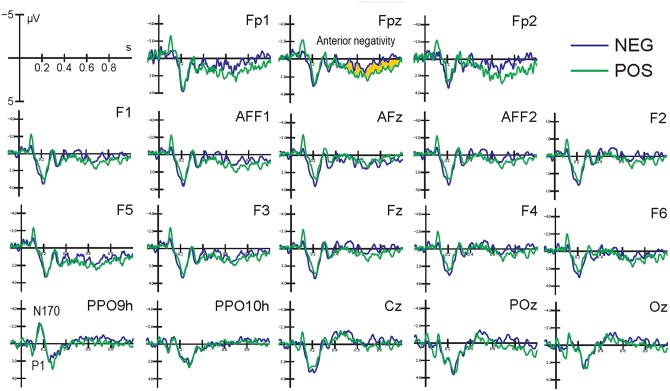
Grand average ERP waveforms recorded at various left and right prefrontal, anterior frontal, dorsolateral prefrontal, inferior frontal, occipito/temporal, occipito/parietal and occipital sites, as a function of prejudice valence (positive vs. negative). A long lasting, late anterior negativity during the processing of negatively biased faces was observed, and no difference at P1 or N170 processing level, due to careful interclass balancing was noted.

Fig 3 shows grand average ERPs recorded at various anterior, central, parietal and posterior sites during the encoding session. Faces belonging to the two classes (positive and negative prejudice) elicited identical ERPs at occipito/temporal sites where sensory and configuration analysis was conducted because of careful balancing of their sensory and semantic characteristics (age, sex, emotional valence of facial expression). However, an increased late negativity during processing of negatively biased faces was observed and quantified at anterior scalp sites.

Anterior negativity (600–800 ms). The ANOVA results showed an effect of prejudice valence (F [1, 11] = 5.482, p<0.05; η2 = 0.33) with larger responses to faces belonging to the NEG (0.64 μV, SE = 0.57) than POS (1.52 μV, SE = 0.78) category.

#### Recognition task

**Fig 4:**
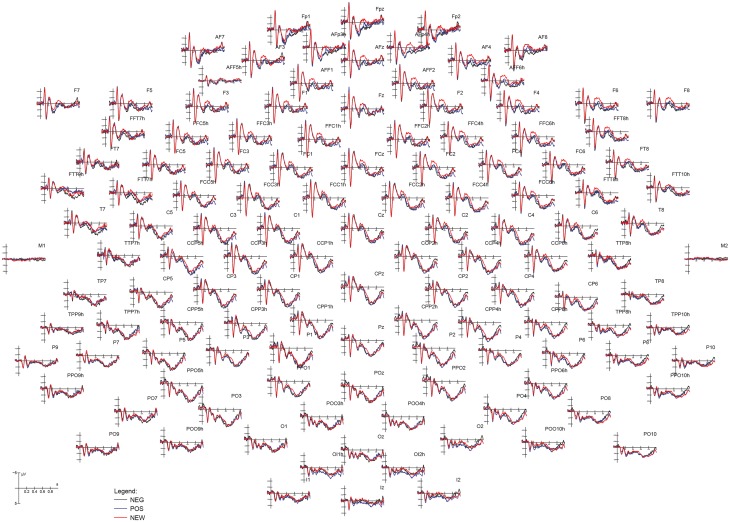
Grand average ERP waveforms recorded from 128 electrode sites during the recognition task. The effect of prejudice affected face processing over prefrontal areas during recognition.

Fig 4 show the ERPs recorded from 128 scalp sites during the recognition task. Large memory effects are visible at anterior (FN400) and parietal sites (Parietal Old/New effect). The effect of prejudice can be appreciated at anterior sites, with a clear gradient in FN400 amplitude from the less familiar to the most familiar items.

**Fig 5:**
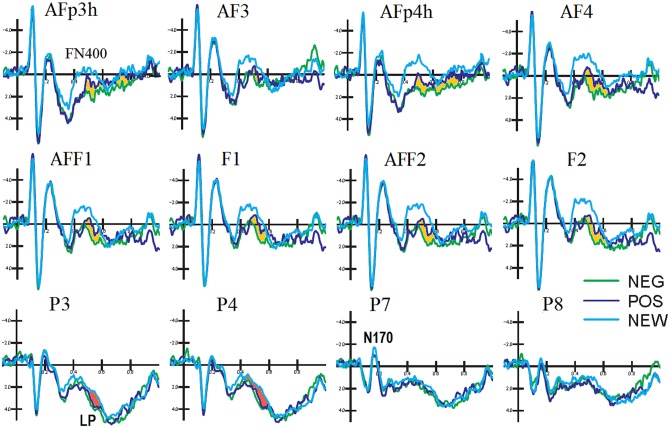
Grand average ERP waveforms recorded at various left and right anterior frontal, dorsolateral frontal, parietal and occipito/temporal sites. A gradient of familiarity is visible at anterior sites, indicating an enhanced processing of new vs. old, and of positive vs. negative faces, possibly negatively correlated to the strength of face memory traces. Recognition of familiar faces (OLD) resulted in the typical parietal old/new effect, in the form of an enhanced late positivity.

FN400 (400–600 ms). The ANOVA performed on FN400 responses recorded at anterior sites revealed a strong effect of face type, with larger potentials to new than old items (F [2, 22] = 7.7973, p<0.005; η2 = 0.41). Post-hoc comparisons indicated greater FN400 responses to NEW (-0.92 μV, SE = 1.74) than POS (0.20 μV, SE = 1.67) faces (p<0.01), and to POS than NEG faces (0.83 μV, SE = 1.59; p<0.01), as evidenced by the ERP waveforms displayed in Fig 5. Furthermore, the significant interaction of face type x hemisphere (F [2, 22] = 4.9964, p<0.01; η2 = 0.31) showed larger FN400s to new faces over the right than left hemisphere (p<0.01), with no other hemispheric asymmetry (NEG LH: 0.70 μV, SE = 1.52; RH: 0.96 μV, SE = 1.67. POS LH: 0.24 μV SE = 1.62; RH: 0.15 μV SE = 1.73; NEW LH: -0.55 μV, SE = 1.68; RH: -1.29 μV, SE = 1.82 μV).

Parietal Old/New effect (LPC 450–550 ms). Face type also significantly affected the parietal LPC which was greater in response to old than new items (F[2, 22] = 5.02, p<0.01; η2 = 0.31), especially over the RH, with no effect of social prejudice on face recollection (NEG LH: 3.12 μV, SE 0.96, RH: 3.16 μV, SE = 0.90; POS LH: 3.12 μV, SE = 1.13; RH: 3.28 μV, SE = 1.14; NEW LH: 2.63μV, SE = 1.07; RH: 2.37 μV, SE = 0.99).

**Fig 6:**
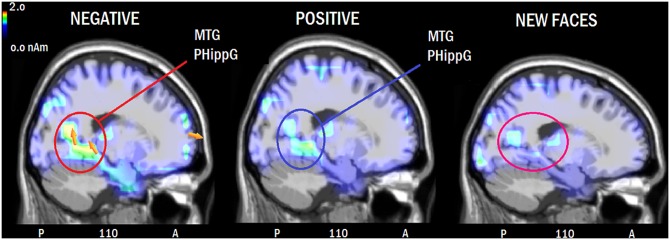
Sagittal views of active sources during processing of negatively-biased, positively-biased and new faces according to swLORETA analysis during the 450–550 ms time window. The different colors represent differences in the magnitude of the electromagnetic signal (in nAm). The electromagnetic dipoles are shown as arrows and indicate the position, orientation and magnitude of dipole modeling solution applied to the ERP waveform in the specific time window. Numbers refer to the displayed brain slice in sagittal view: A = anterior, P = posterior. The images highlight the strong activation of the left medial temporal and parahippocampal gyri during memory recall of faces associated with a negative prejudice.

**Fig 7:**
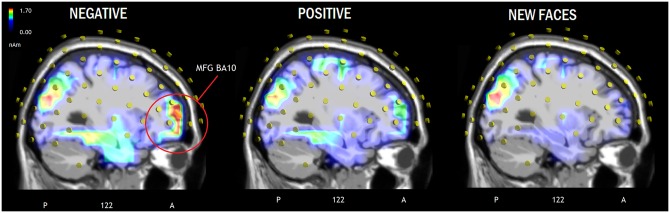
Sagittal views of active sources during processing of negatively-biased, positively-biased and new faces according to swLORETA analysis during the 450–550 ms time window. The images highlight the strong activation of the left medial frontal gyrus during memory recall of faces associated with a negative prejudice.

LORETA source reconstruction. To identify the intracranial neural generators of surface potential, an inverse solution swLORETA was applied to ERPs recorded during the recognition task for the processing of the 3 types of faces (NEG; POS, NEW) in the time window corresponding to the recollection (LPC) and familiarity (FN400) effects (450–550 ms). Table 3 shows the Talairach coordinates (in mm) corresponding to the active sources, in addition to their magnitude (in nAm). Recall of faces (old vs. new) was associated with activity in the right superior frontal cortex, left medial temporal gyrus (BA 39) and right fusiform gyrus (FG, BA37, also called “fusiform face area”). However, recall of negative faces was uniquely associated with activations in the left superior frontal gyrus (BA10), left posterior cingulate cortex (BA20) and left parahippocampal gyrus (BA30) of the limbic system (see Figs 6 and 7 for a comparison between the three conditions). The right cingulate cortex (BA30/32) and right occipital areas, such as the inferior occipital gyrus (BA18/19) and the right precuneus, were commonly activated during the perception of old and new faces regardless of prejudice valence, therefore indicating an effect of face processing and not memory.

**Table 3:** Talairach coordinates (in mm) corresponding to intracranial generators explaining the surface voltage recorded during the 450–550 ms time window during processing of various face types in the recognition task. Magn. = Magnitude in nAm; H = hemisphere, BA = Brodmann areas.

**Negative Bias**							
**Magn.**	**T-x [mm]**	**T-y [mm]**	**T-z [mm]**	**Hem.**	**Lobe**	**Gyrus**	**BA**
2.17	40.9	-86.4	-12.4	Right	Occ	InfOcc OFA	18
2.06	-28.5	54.4	15.9	**Left**	**Front**	**SupFr**	**10**
1.95	-8.5	64.4	16.8	**Left**	**Front**	**SupFr**	**10**
2.03	50.8	-55	-17.6	Right	Temp	Fus, FFA	37
1.95	-48.5	-57.9	5.6	Left	Temp	MTG	39
1.82	1.5	57.3	-9	Right	Front	MFG	10
1.81	-18.5	-36.6	-1.3	**Left**	**Limbic**	**Phippoc**	**30**
1.79	-18.5	-58.9	14.5	**Left**	**Limbic**	**pCingulate**	**20**
1.79	1.5	-48.7	15.3	Right	Limbic	pCingulate	30
**Positive Bias**							
2.09	40.9	-75.2	-19.1	Right	Occ	InfOcc, OFA	18
1.98	50.8	-55	-17.6	Right	Temp	Fus, FFA	37
1.84	-48.5	-69	13.6	Left	Temp	MTG	39
1.90	21.2	56.3	-1.6	Right	Front	SupFr	10
1.83	11.3	-40.6	34	Right	Limbic	Cingulate	31
1.89	21.2	-82.1	39.5	Right	Par	Precuneus	19
**New Faces**							
2.89	40.9	-86.4	-12.4	Right	Occ	Precuneus	18
2.16	-38.5	-72	40.3	Left	Par	Precuneus	19
2.13	21.2	-82.1	39.5	Right	Par	Ling (OFA)	19
1.91	-8.5	-96.5	-13.1	Left	Occ	Ling (OFA)	17
1.82	11.3	-41.5	42.9	Right	Limbic	Cingulate	31

## Discussion

The aim of the study was to investigate the effect of negative prejudicial information associated with a face on memory processes. Therefore, we presented fictional information relative to 200 male and female faces during an encoding task. After approximately 30 minutes, memory for faces was tested with an old/new paradigm involving 100 new faces. Correct response percentage was approximately 75%, and it was easier to correctly recognize a face as new (87%) than old (69%). However, RTs were much faster to old than new faces, regardless of prejudice valence. Overall, the prejudice did not improve recollection of negative vs. positive faces after 30 minutes in the encoding session. The lack of effect at the behavioral level is consistent with other studies that did not find an effect of prejudicial bias on old/new face recognition [34,35]. Therefore, the hypothesis can be extended; negative bias enhanced memory consolidation by involving emotional associative areas. In a very recent and interesting fMRI study [39] during encoding of emotionally arousing information, a robust increase in effective connectivity from the amygdala to the hippocampus was observed, regardless of stimulus valence (both positive and negative).

Indeed, stimuli of our study were created in a way that positive bias significantly modulated face trustworthiness, thereby improving the ability to recall faces and their learning context. However, ERP data provided evidence of a dissociation between familiarity and recollection, with an effect of valence on FN400 but not LPC amplitude, in ERPs recorded during the recognition test.

Because faces were carefully balanced for sensory and perceptual characteristics as well as for sex, age, and facial expression (see [40] for the effects of face age on N170 amplitude), prejudicial bias did not affect the amplitude of N170 response, which was thought to index the encoding of structural face properties [41,42]. The first effect of prejudice valence (during the encoding session) was found at the anterior frontal and prefrontal areas, between 600 and 800 ms after face presentation. It is interesting to note that ERPs were time locked to face onset because the verbal description (fictional story) was presented 3.5. sec in advance. Therefore, this effect (increase in anterior negativity) might index an additional processing of face-related information associated with a negative context (negative social traits). The role of the prefrontal cortex in episodic memory has been widely documented [43], whereas anterior negativity has been clearly related to stimulus complexity, load and working memory span [44,45]. Our data suggest that negative faces were encoded more deeply (as typical of aversive stimuli), which explains the difference in FN400 observed in the recognition test for NEG vs. POS faces. Indeed, anterior FN400 was both greater to new than old items, as expected based on the ERP literature on FN400 familiarity effects [31], and greater to POS than NEG stimuli as well. This finding might indicate that POS stimuli were less familiar than NEG stimuli because they were more superficially coded during the encoding session. Specifically, a dissociation was found between familiarity and recollection (indexed by LPC, i.e., the parietal/old new effect). Indeed, LPC was larger in response to old than new items, but it was not modulated by bias valence, which highly correlated with behavioral data. A similar correlation between behavioral performance and the parietal ERP effect was found in an ERP study involving memory for words and their source with an old/new paradigm [46]. Again, in strong agreement with our study comes a recent ERP investigation on memory [47] in which it was compared neural processing of faces associated with negative, positive or neutral contexts (cheating, cooperation, or neutral behavior in a social-dilemma game). Quite similarly to our study, it was found a frontal correlate of context valence and a parietal old-new response (which was not modulated by valence) in response to the faces presented in the memory test. Furthermore, there was no effect on early correlates of face processing such as the N170, which is consistent with the idea that the N170 is an index of perceptual face processing, not being much affected by memory factors. Overall, the present ERP pattern of results agrees with current knowledge about recollection/familiarity dissociation [48,27,28]and provides interesting information about the processing of aversive social information, which implicitly affected stimulus familiarity.

Our source reconstruction data obtained by applying swLORETA to scalp potentials recorded during the maximum amplitude of familiarity and recalling effects (450–550 ms) support our hypothesis that NEG faces were coded more robustly during the learning phase (as also evidenced by the enlarged prolonged anterior negativity elicited by NEG vs. POS faces during that experimental session).

Overall, remembering faces (old vs. new contrast) was associated with significant source activity in the right superior frontal cortex (BA10). Right prefrontal activity has been suggested to characterize episodic retrieval mode by many influential studies [49,50]. Another common activation by old but not new faces (regardless of valence) occurred in the left medial temporal gyrus (MTG, BA39). Neuroimaging studies also in addition to studies on amnesic patients have revealed that the regions of the medial temporal lobe have a crucial role in episodic memory and recognition memory [51], [52,53,54]. The left hemispheric asymmetry of MTG for episodic memory for people is consistent with previous investigations, suggesting a left hemisphere role in memory for people [55]. Old (vs. new) face processing was also associated with strong activity in the right fusiform area of the temporal gyrus, also named FFA. The role of this region in face configuration processing is well documented [56], in addition to the involvement of the right prefrontal cortex in face processing [57–60]. Because both areas were only activated during processing of old (and not new) faces, as the areas may be possible neural loci for storing face-related episodic memory information.

Remembered (OLD) and non-remembered (NEW) faces shared the activation of lateral occipital areas (BA18/19), possibly indicating the activity of the occipital face area (OFA) during face processing. Similar to FFA, OFA would also be involved in face configuration analysis [56] but would be more responsive to local details (e.g., the eyes, the mouth, the nose [61] than the holistic pattern). Remembered and non-remembered faces also shared the activation of the posterior cingulate cortex (involved in the emotional processing of visual images [62] and empathy [63] and the right precuneus. In regard to the effect of bias prejudice, processing of NEG vs. POS faces was associated with increased activity in the left superior frontal gyrus (BA10), left posterior cingulate cortex (BA20) and left parahippocampal gyrus (BA30) of the limbic system. Overall, brain activation was higher during processing of NEG vs. POS faces, therefore indicating a stronger recognition response to aversive (than positive) social information [10–13]. Indeed, the left lateral prefrontal cortex is involved in the processing of familiarity [64] and episodic recall [65]; whereas parahippocampal regions are thought to be involved in the recovery of memory contextual information [66–68]. Overall, a dissociation was found between face recollection and accuracy as evidenced by the parietal old/new effect, and face familiarity (indexed by FN400 component) showed a reduced response to NEG than POS or NEW faces. The enhanced anterior negativity during coding of NEG vs. POS faces in the learning session is consistent with FN400 behavior in the recognition session, as well as with the finding of enhanced brain activation in prefrontal, parahippocampal and limbic areas during recall of negative faces.

The results of our study, and particularly that negative social bias did not modulate perceptual N170 response to faces in the recognition task are in apparent contradiction with some ERP investigations showing how affective information and context can indeed modulate N170 component. However it should be considered that a crucial difference exists between our study and other experimental designs. In these studies, the context and prejudicial information was co-existent or pre-existent, or even rooted in subjects’ mind/brain while they perceived faces (thus affecting face processing), while in our study the random association between a face and a verbal description had to be specifically remembered, otherwise it was not available. For example, in a study by Hietanen and Astikainen (2013) [69], in which ERPs were recorded to happy and sad facial expressions preceded by positive and negative scenes (which acted as primes) faces were presented 300 ms after the prime, therefore the context was almost simultaneous to the faces. In another investigation by Ibanez et al.’s (2010) [70] N170 response was influenced by IAT in-group vs out-group effects (i.e., social prejudices possessed by subjects), therefore each face carried the prejudicial information (race), and nothing had to be remembered. Again, in another ERP study by Martínez-Galindo and Cansino (2015) [71], in which participants performed a betting-game task while the faces of their virtual opponents were presented in each trial, the N170 component was more negative for faces encoded in positive contexts (while winning a bet) than for those encoded in non-emotional contexts, but the general effect of context was not face-specific, and, according to the authors, it could not be excluded that the context might have modified the overall cerebral arousal. This effect is impossible in the present study, in that positive and negative prejudices were presented during the same experimental run, randomly intermixed. Last but not least, it would be mentioned that, in our knowledge, the memory literature about old/new effects has not shown a memory modulation for N170 response.

Our findings are strongly in agreement with previous fMRI data showing the role of the medial frontal cortex in representing social information referred to others, particularly outgroup stereotyping and prejudice [72]. They are also in absolute agreement with a quite recent ERP investigation testing memory for objects and showing a dissociation between FN400 and late old/new parietal effects, thought to index stimulus familiarity and memory recollection, respectively. In this interesting study a familiarity effect was found at mid-frontal level, which was dissociated by the (missing) parietal effect and failure in recollection [73]. This finding may further explain why, in our study, brain signatures of memory for negatively biased faces (indexed by FN400 in the recognition task and prefrontal activity in the learning phase) were dissociated by performance in recollection and LP amplitude, with specific regard to the lack of positive/negative gradient.

### Study Limits

It would have been interesting to compare the coding of faces provided with valenced vs. neutral information, or valenced vs. without any additional information, and these comparisons should be investigated in future studies.

Currently, we cannot exclude an effect of prejudicial bias on memory recollection (in terms of accuracy or brain activation) in long-term memory (LTM). Indeed, in our study memory retrieval occurred 30 minutes after the learning session, whereas the long term effects of aversive episodic memory would have been observed, for example after a week [7] or 6 months after learning. This issue certainly deserves further investigation. Furthermore, the task was probably very difficult, because of the large number of faces to be remembered.

Because of the intrinsic nature of bioelectrical potentials, it is almost impossible to detect the activity of small subcortical nuclei, such as the amygdala, in source reconstruction analysis. This factor does not exclude its crucial role in aversive memory consolidation that has been widely shown in neurometabolic and cell recording neurophysiological studies.
